# Does early resection of presumed low-grade glioma improve survival? A clinical perspective

**DOI:** 10.1007/s11060-017-2418-8

**Published:** 2017-04-11

**Authors:** Maarten M. J. Wijnenga, Tariq Mattni, Pim J. French, Geert-Jan Rutten, Sieger Leenstra, Fred Kloet, Martin J. B. Taphoorn, Martin J. van den Bent, Clemens M. F. Dirven, Marie-Lise van Veelen, Arnaud J. P. E. Vincent

**Affiliations:** 1 Department of Neuro-Oncology, Erasmus MC Cancer Institute, Wytemaweg 80, 3015CN Rotterdam, The Netherlands; 2 Department of Neurosurgery, Erasmus MC Cancer Institute, Wytemaweg 80, 3015CN Rotterdam, The Netherlands; 3 Department of Neurosurgery, Elisabeth-TweeSteden Hospital, Tilburg, The Netherlands; 4 Department of Neurosurgery, Haaglanden Medical Centre, The Hague, The Netherlands; 5 Department of Neurology, Haaglanden Medical Centre, The Hague, The Netherlands

**Keywords:** Diffuse low-grade glioma, Wait-and-scan, Biopsy, Resection, Survival

## Abstract

Early resection is standard of care for presumed low-grade gliomas. This is based on studies including only tumors that were *post-surgically confirmed* as low-grade glioma. Unfortunately this does not represent the clinicians’ situation wherein he/she has to deal with a lesion on MRI that is *suspect* for low-grade glioma (i.e. without prior knowledge on the histological diagnosis). We therefore aimed to determine the optimal initial strategy for patients with a lesion *suspect* for low-grade glioma, but not histologically proven yet. We retrospectively identified 150 patients with a resectable presumed low-grade-glioma and who were otherwise in good clinical condition. In this cohort we compared overall survival between three types of initital treatment strategy: a wait-and-scan approach (n = 38), early resection (n = 83), or biopsy for histopathological verification (n = 29). In multivariate analysis, no difference was observed in overall survival for early resection compared to wait-and-scan: hazard ratio of 0.92 (95% CI 0.43–2.01; p = 0.85). However, biopsy strategy showed a shorter overall survival compared to wait-and-scan: hazard ratio of 2.69 (95% CI 1.19–6.06; p = 0.02). In this cohort we failed to confirm superiority of early resection over a wait-and-scan approach in terms of overall survival, though longer follow-up is required for final conclusion. Biopsy was associated with shorter overall survival.

## Introduction

Diffuse low-grade gliomas (LGGs) are primary brain tumors that, due to their infiltrative nature, cannot be fully eradicated by resection, chemotherapy, radiation, or a combination of these regimens. Most LGGs will gradually evolve into higher-grade gliomas and almost all patients will ultimately die from the disease [1, 2].

The typical LGG patient presents with a first epileptic seizure and a lesion on MRI that is suspect for a LGG (isointense to hypointense and non-enhancing on T1-weighted images; hyperintense on T2-weighted and fluid attenuated inversion recovery (FLAIR) images) [3]. Consequently, in combination with clinical parameters, but yet without confirmed histology, physicians have to decide on a treatment strategy. They can opt for a wait-and-scan policy, take a biopsy for histopathological verification, or opt for immediate resection. Treatment strategy is patient dependent, influenced by the clinical condition of the patient, seizure control, size of the tumor and resectability of the tumor [4]. An initial wait-and scan approach is usually followed by resection or biopsy at the time the lesion starts to show growth or enhancement on MRI, or when clinical deterioration occurs. Resection and biopsy can either be followed by a wait-and-scan policy, radiotherapy, chemotherapy, or a combination of the latter two [4] Both chemotherapy and radiotherapy have been extensively investigated in randomized controlled trials [5–11]. Controlled trials exploring the role and timing of surgery are lacking and, therefore, surgery for LGG has been controversial for many years.

In the past an initial wait-and-scan approach was advocated, since LGGs tend to grow slowly and patients usually present with controllable seizures as the only clinical symptom [12–14]. However, in the last 20 years, general opinion has shifted and early maximal resection is now widely accepted for patients with LGG-like lesions that are eligible for resection. Indeed, multiple retrospective studies showed that a more extensive resection is associated with a marked improvement of overall survival [15–23]. Also, a study in Norway showed that early resection significantly improves overall survival compared to a biopsy with a subsequent watch-and-wait period [24, 25]. This growing bulk of evidence, although retrospective, has logically resulted in early maximal resection being standard of care and being incorporated in international guidelines nowadays [4].

However, we have to bear in mind that these retrospective studies are subject to at least some form of selection and indication bias. Firstly, these studies excluded the non-enhancing presumed LGGs that are diagnosed as a higher grade after early surgery. Secondly, these studies discarded the presumed LGGs that progressed to a higher grade during the wait-and-scan period. Thirdly, these studies included patients with confirmed LGG, but with preoperative enhancement on MRI, which is usually not a presumed low-grade glioma [26–28]. Possibly there is also indication bias present in these studies; the physicians choice for initial treatment is potentially influenced by factors that also have impact on prognosis itself [29]. In conclusion, the cohorts used in these previous studies are not entirely representative for the daily clinical situation in which physicians are confronted with a LGG-like lesion on MRI without histological confirmation and, consequently, in combination with clinical parameters, have to decide for an initial treatment strategy. Therefore, a study with patient selection based solely on preoperative clinical and imaging characteristics is more clinically relevant and can add significant evidence to support current daily clinical practice. A prospective trial is warranted but is unlikely to be conducted due to the duration of such a study (median survival of ≥15 years in oligodendroglioma subtype [13]), ethical considerations raised by physicians who strongly believe in early resection, as well as obtaining patients’ consent to randomize between radically different treatment strategies.

In this retrospective study we approached the issue of treatment strategy from a more clinical and preoperative point of view and selected patients with a resectable LGG-like lesion based on diagnostic imaging and not on histopathological confirmation. We included those patients that we retrospectively consider equally eligible for either a wait-and-scan approach, a biopsy for histological verification, or early resection as initial treatment strategy; i.e. patients had to have limited neurological deficits that allowed a wait-and-scan strategy but also a LGG-like lesion that was eligible for extensive resection (estimation of at least 80% volume reduction possible, with use of current available techniques like awake surgery). In this manner we eliminated selection bias by histology and we avoided selection bias on indication as much as possible.

The aim was to determine the optimal initial treatment strategy for a resectable, *presumed* low-grade glioma by comparing overall survival between wait-and-scan, early resection and a biopsy approach.

## Methods

### Patient selection

Three large neurosurgical institutions participated in this cohort study, together serving a population of 6.5 million people in the southwest of the Netherlands. The institutions involved were the Erasmus MC Cancer Institute in Rotterdam (EMC), Elisabeth-TweeSteden Hospital in Tilburg (ETZ), and Medical Centre Haaglanden in The Hague (MCH).

We identified patients with a presumed LGG (LGG-like lesion) that were retrospectively eligible for either initial treatment strategy: i.e. initial wait-and-scan approach, biopsy for histopathological verification, or immediate resection. Well-established prognostically favorable radiological and clinical characteristics were used as inclusion criteria [30–32]. Radiological criteria were: supratentorial location of lesion, no contrast enhancement, no midline shift, maximal diameter <6 cm, sharply defined borders, and no involvement of corpus callosum, basal ganglia or thalamus. Clinical criteria were: age ≥ 18 years, Karnofsky Performance Status (KPS) >70, neurologically stable (with only epilepsy or minimal neurological deficits), and no dexamethasone dependency. Patients with active synchronous cancer of other origin were excluded.

**Fig. 1:**
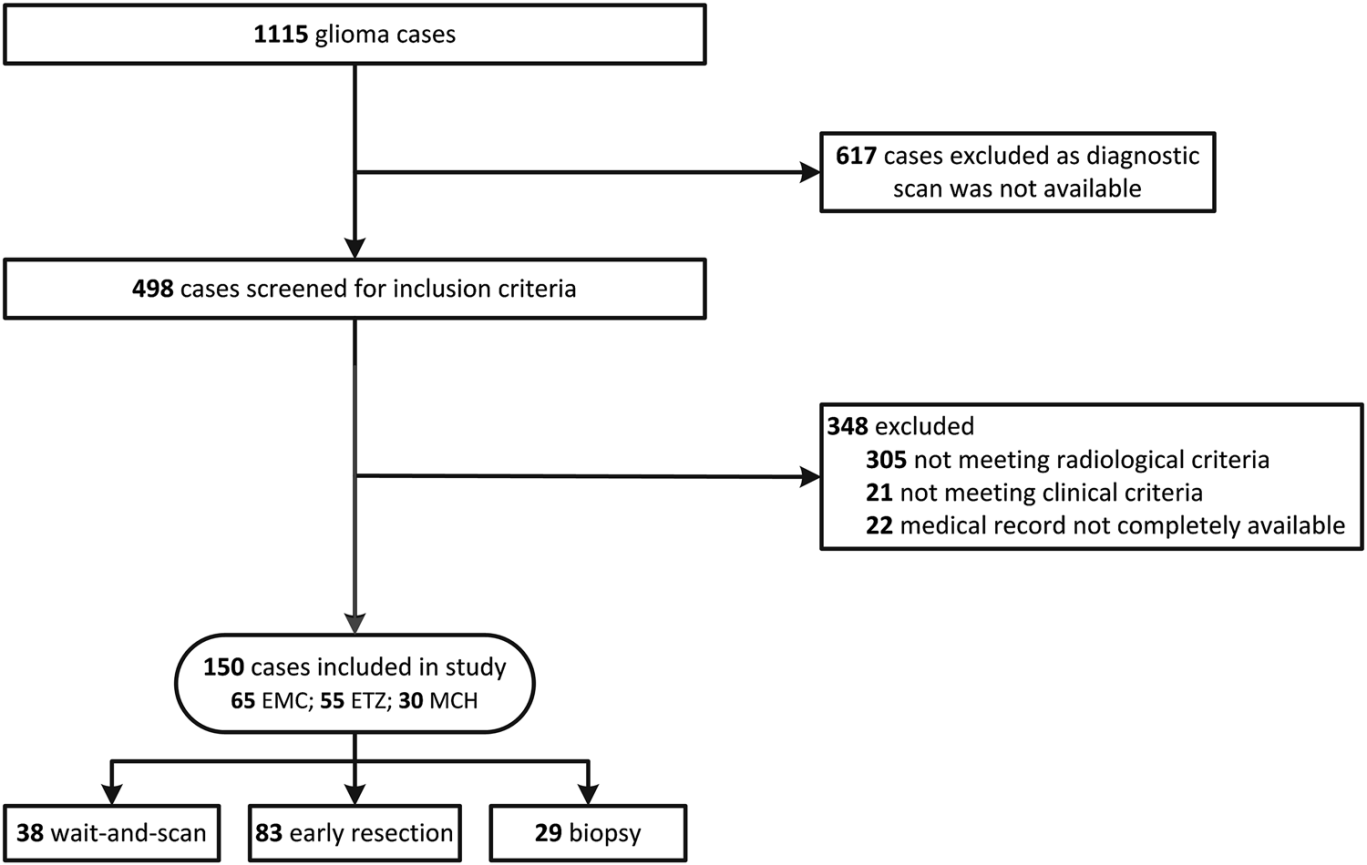
Consort flow diagram of patient inclusion. Of the 1115 glioma patients identified with a search in the digital patient archives, a diagnostic scan could be retrieved for 498 of them. Of these, 305 were excluded as they did not meet radiological criteria, 21 did not meet clinical criteria, and for 22 the complete medical record was not available. A total of 150 cases remained for analysis

To identify glioma patients, the digital archives of patient letters were searched for all neurological and neurosurgical patients registered 1990–2010 in EMC, 1996–2010 in ETZ, and 1992–2010 in MCH. In this first selection, high-grade gliomas were included so as not to exclude patients who progressed to a higher grade during a wait-and-scan period. In this search, of the 1115 glioma patients identified, a diagnostic scan could be retrieved for 498 of them, while the other part mainly originated from the pre digital era and was not available anymore. All diagnostic scans were reviewed to check if they met the criteria for (a) the radiological diagnosis of a low-grade glioma and (b) for the feasibility of an extensive resection (estimation of at least 80% volume reduction possible with modern surgical techniques like awake surgery) by a single neurosurgeon (AJPEV). Tumor near eloquent location was not an exclusion criterion per se, since the vast majority is eligible for resection with modern surgical techniques. The reviewing neurosurgeon has more than 10 years’ experience in awake surgery and was blinded for clinical information such as initial treatment strategy, the histopathological diagnosis and outcome. Of these 498 patients, 348 were excluded: 305 did not meet the radiological criteria, 21 did not meet the clinical criteria, and for 22 the complete medical records were not available. Eventually, 150 patients remained for analysis. An overview of the selection procedure is shown in Fig. 1.

### Study variables

Baseline characteristics of the patients were collected from the medical records and diagnostic scan; i.e. initial treatment strategy, gender, age, KPS, presenting symptom, tumor location, mean tumor diameter and tumor eloquence; eloquence was graded with the criteria of Chang et al. [32].

Three types of initial treatment strategy were compared: initial wait-and-scan strategy after radiological diagnosis, early resection, and initial biopsy procedure for histological verification. Treatment decisions were based on local, national and international guidelines in each individual centre at that time, by an experienced multidisciplinary team. Postoperative characteristics were also collected: first histology and grade, total number of resections, type of surgery (awake vs. general anesthesia), subsequent strategy after early resection, or biopsy and administration of radiotherapy and/or chemotherapy. Because postoperative MRI or CT scans were not available for most operated patients, the extent of resection could not be reliably investigated. In most tumors, molecular markers were not available and therefore not included in the analysis.

### Outcome measure

The primary outcome measure was overall survival (OS), which was defined as the time between the diagnostic scan and death. All included patients were followed until death or censored at the date of last follow-up. Date of death was provided by patient records or the Municipal Personal Records Database.

### Statistical analysis

All analyses were performed using R (3.1.3) and RStudio (0.99.486). Categorical data were analyzed with Pearson’s Chi square test or Fisher’s exact test when assumptions of the Chi square test were violated. Continuous data were analyzed with a Kruskal–Wallis test. Overall survival is shown in Kaplan–Meier plots (ggplot2 package in R). Univariate and multivariate analyses were performed using a Cox proportional hazard model (survival CRAN package in R). All calculations were two-sided tests, with a p value <0.05 considered as statistically significant.

### Ethics and approvals

Need for informed consent was waived by the Medical Ethical Committee of Erasmus MC, Rotterdam.

## Results

The medical records and diagnostic scans of 498 identified glioma patients were screened with our selection criteria to select patients with a resectable lesion and relatively favorable prognostic characteristics. A total of 150 patients with a resectable presumed LGG were included (Fig. 1). The initial treatment strategy was either an initial wait-and-scan approach (n = 38), a biopsy for histopathological verification (n = 29), or early resection (n = 83). Median follow-up was 7.1 years (25–75% interquartile range: 5.4–9.8 years). Baseline characteristics were equally distributed between treatment groups, except for tumor location in eloquent area (15.8% in wait-and-scan vs. 10.3% in biopsy and 32.5% in early resection; p = 0.02) (Tables 1, 2).

**Table 1:** Patient characteristics at baseline

Characteristic	Treatment strategy			P
	Wait-and-scan	Early resection	Biopsy	
	(N = 38)	(N = 83)	(N = 29)	
	N (%)^a^	N (%)^a^	N (%)^a^	
Gender				0.14
Male	25 (65.8)	40 (48.2)	18 (62.1)	
Female	13 (34.2)	43 (51.8)	11 (37.9)	
Age in years				
Median (IQR^b^)	38 (16.3)	39 (14.6)	41 (21.4)	0.14
<40	23 (60.5)	46 (55.4)	13 (44.8)	0.43
KPS at diagnosis				0.28
100	37 (97.4)	77 (92.8)	24 (82.8)	
90	1 (2.6)	5 (6.0)	4 (13.8)	
80	0 (0.0)	1 (1.2)	1 (3.4)	
Presenting symptom				0.56
Epilepsy	35 (92.1)	71 (85.5)	23 (79.3)	
Cognitive disorder	0 (0.0)	1 (1.2)	0 (0.0)	
Hemiparesis	0 (0.0)	1 (1.2)	2 (6.9)	
Speech disorder	0 (0.0)	1 (1.2)	1 (3.4)	
Incidental finding	3 (7.9)	6 (7.2)	3 (10.3)	
Headache	0 (0.0)	3 (3.6)	0 (0.0)	
Tumor location				0.94
Frontal	18 (47.4)	46 (55.4)	13 (44.8)	
Temporal	7 (18.4)	14 (16.9)	6 (20.7)	
Parietal	5 (13.2)	11 (13.3)	4 (13.8)	
Occipital	1 (2.6)	1 (1.2)	0 (0.0)	
Insula	7 (18.4)	11 (13.3)	6 (20.7)	
Eloquent area				0.02
Yes	6 (15.8)	27 (32.5)	3 (10.3)	
No	32 (84.2)	56 (67.5)	26 (89.7)	
Tumor diameter (mm)				
Median (IQR^b^)	39.5 (12.0)	41.0 (16.5)	41.0 (10.0)	0.67

^**a**^Data are numbers (%) unless indicated otherwise

^b^Interquartile range (25–75%)

**Table 2:** Tumor and treatment characteristics of the three groups

Characteristics	Treatment strategy			P
	Wait-and-scan	Early resection	Biopsy	
	(N = 38)	(N = 83)	(N = 29)	
	N (%)^a^	N (%)^a^	N (%)^a^	
Number of resections				<0.001
Zero	8 (21.1)	0 (0.0)	14 (48.3)	
One	25 (65.8)	47 (56.6)	10 (34.5)	
Two	5 (13.2)	34 (40.9)	5 (17.2)	
Three	0 (0.0)	2 (2.4%)	0 (0.0)	
Procedure of first surgery				0.36
Awake	14 (46.7)	38 (45.8)	4 (26.7)	
General anesthesia	16 (53.3)	45 (54.2)	11 (73.3)	
Subsequent treatment after initial resection or biopsy^b^				<0.001
Wait-and-scan	Not applicable	53 (66.3)	3 (11.5)	
Radiotherapy	Not applicable	26 (32.6)	22 (84.6)	
Other	Not applicable	1 (1.3)	1 (3.8)	
Ever radiotherapy				0.01
Yes	28 (73.7)	57 (68.7)	28 (96.6)	
No	10 (26.3)	26 (31.3)	1 (3.4)	
Ever chemotherapy				0.02
Yes	23 (60.5)	30 (36.1)	16 (55.2)	
No	15 (39.5)	53 (63.9)	13 (44.8)	
First histology				0.01
Astrocytoma	16 (42.1)	40 (48.2)	22 (75.9)	
Oligodendroglioma	12 (31.6)	30 (36.1)	7 (24.1)	
Oligo-astrocytoma	9 (23.7)	13 (15.7)	0 (0.0)	
Not yet known	1 (2.6)	0 (0.0)	0 (0.0)	
Grade				0.04
II	28 (75.7)	74 (89.2)	28 (96.6)	
III	7 (18.9)	9 (10.8)	1 (3.4)	
IV	2 (5.4)	0 (0.0)	0 (0.0)	

^a^Data are shown as numbers (%)

^b^Treatment after intervention is shown for the groups in which the initial strategy was immediate resection or biopsy

Median time between diagnostic scan and intervention was 35.4 months in the wait-and-scan group, 0.8 months in the biopsy group, and 2.9 months in the early resection group. In 80% (n = 66) of patients in the early resection group surgery was performed within 6 months after the diagnostic scan. Of the remaining 20% (n = 17), all received surgery within 1 year, without any sign of tumor growth, enhancement or clinical deterioration at time of surgery. In these latter patients the physicians’ initial choice of treatment was an early resection. However, the time between diagnosis and resection was ≥6 months, mainly due to practical reasons; either because referral from the diagnosing centre to neurosurgical centre was delayed, or due to patients’ doubts about the treatment strategy. Nevertheless, these 17 patients were not excluded from the analysis as the actual initial choice of treatment was early resection and the intervention took place when there was still no sign of clinical deterioration, tumor growth or contrast enhancement on the control MRI. However to rule out bias, a sensitivity analysis was performed with the exclusion of these patients (see below).

In the group with wait-and-scan as initial treatment strategy, 79% of the patients eventually underwent a resection during follow-up. In these patients, surgery was initiated because of signs of growth or enhancement on follow-up imaging. There were no patients with uncontrolled seizures in the wait-and-scan group, nor was this a reason for surgery during follow-up. In the biopsy group, 51.7% eventually underwent resective surgery. Distribution of the postoperatively obtained tumor characteristics (histology and grade) differed between the groups: the biopsy group consisted of more astrocytomas (75.9 vs. 42.1% in wait-and-scan and 48.2% in early resection; p = 0.01) and the wait-and-scan group consisted of more gliomas of higher-grade (24.3 vs. 10.8% in resection and 3.4% in biopsy; p = 0.04).

**Fig. 2:**
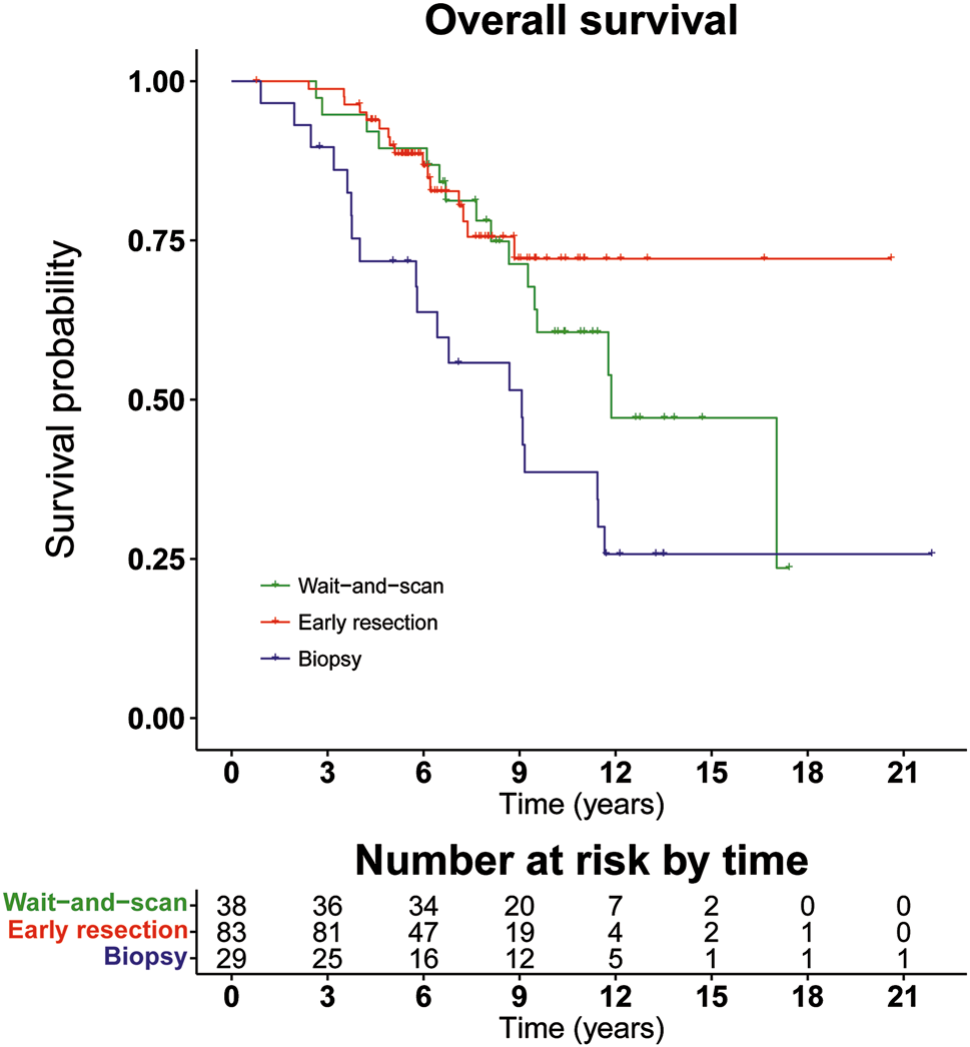
Kaplan–Meier plot showing the overall survival per treatment strategy. The p value is calculated by the log-rank test including all three treatment groups. In the lower table, data indicate the numbers of patients at risk at the given time

Median OS in the early resection group was not reached and showed no significant difference (p = 0.42) from the wait-and-scan group in which the median OS was 11.9 years (95% CI 9.5–∞) (Fig. 2). However, the median OS of 9.1 years (95% CI 5.8–∞) in the biopsy group was significantly shorter compared to both the wait-and-scan and early resection group (log-rank test; p = 0.04 and p = 0.001, respectively) (Fig. 2).

In the univariate analysis, histology, grade, tumor location and tumor eloquence also had a significant impact on OS (Table 3) and were, therefore, included in the multivariate Cox regression. In this multivariate analysis, the difference in OS remained with a hazard ratio (HR) of 2.53 (95% CI 1.1–6.1; p = 0.04) (Table 3) for the biopsy group compared to wait-and-scan, whereas no difference was observed for early resection compared to wait-and-scan (HR 0.92; 95% CI 0.43–2.01; p = 0.85).

**Table 3:** Univariate and multivariate analysis of overall survival using the Cox proportional hazards model

Variable	Univariate		Multivariate	
	HR (95% CI)	P	HR (95% CI)	P
Treatment				
Wait-and-scan	1		1	
Early resection	0.72 (0.36–1.46)	0.37	0.92 (0.43–2.01)	0.85
Biopsy	2.04 (1.05–3.99)	0.04	2.69 (1.19–6.06)	0.02
Eloquency				
Yes	1		1	
No	2.36 (1.00–5.54)	0.05	1.41 (0.57–3.49)	0.46
Histology				
Astrocytoma	1		1	
Oligodendroglioma	0.40 (0.19–0.87)	0.02	0.49 (0.22–1.09)	0.08
Oligo-astrocytoma	1.10 (0.52–2.30)	0.81	1.34 (0.58–3.11)	0.49
Grade				
>II	1		1	
II	0.49 (0.24–0.98)	0.04	0.40 (0.17–0.93)	0.03
Tumor location				
Frontal	1		1	
Temporal	3.40 (1.65–6.88)	<0.001	3.49 (1.66–7.35)	<0.001
Parietal	1.90 (0.88–4.24)	0.10	1.60 (0.72–3.56)	0.25
Occipital	0.00 (0.0–∞)	0.99	0.00 (0.00–∞)	0.99
Insula	2.30 (1.03–4.94)	0.04	2.79 (1.21–6.40)	0.02

*HR* hazard ratio, *∞* infinite

A sensitivity analysis was also performed excluding those patients in the early resection group that did not undergo a resection within 6 months after diagnosis. No difference in OS was found for early resection compared to wait-and-scan with a HR of 0.70 (95% CI 0.33–1.46; p = 0.34) in univariate analysis and 0.83 (95% CI 0.37–1.86; p = 0.65) in multivariate analysis (including also histology, grade, and tumor location/eloquence as variables). A significant difference in OS remained for biopsy versus wait-and-scan, with a HR of 2.03 (95% CI 1.03–3.99; p = 0.04) in univariate analysis and 2.82 (95% CI 1.24–6.43; p = 0.01) in multivariate analysis.

## Discussion

Early maximal safe resection is considered standard of care for presumed LGG. Evidence to support this approach is mainly derived from retrospective studies; clear evidence from prospective trials for this early aggressive surgical approach is not available. Arguments in favor of early resection include uncertainty about the radiological diagnosis, the assumption that resection will postpone malignant transformation and will improve overall survival [20, 26]. Indeed, several retrospective studies affirm the hypothesis that extensive resection for LGG improves overall survival [15–23]. Concerns that resection in a later stage of the disease could technically be more difficult and induce malignant transformation are not unimaginable. In the light of these concerns and the association between extent of resection and overall survival, one may argue that the attempt for an extensive resection should be made as early as possible. These concerns that extent of resection can be influenced by timing of surgery in LGG have not been investigated by any study so far. Timing of treatment itself without incorporation of extent of resection has been studied before however. In a study by Jakola et al., a unique situation in Norway was studied wherein treatment outcome was compared between two centers: one centre favoured biopsy with subsequent watchful waiting strategy and the other centre early resection. The early resection strategy in one centre was clearly associated with a longer overall survival [24, 25]. For several years now, after years of controversy, the approach of early maximal resection logically is incorporated in treatment guidelines for LGG. However, despite current guidelines, we have to bear in mind those studies were biased by histopathological diagnosis as inclusion criterion. This selection is actually not representative for the daily clinical setting whereby an initial treatment decision is based on imaging and patient characteristics. Although a prospective trial is the golden standard to clarify this issue, this is generally considered to be infeasible. We therefore tried to confirm the observations and assumptions from earlier studies that early resection prolongs overall survival in a cohort that more closely mimics daily clinical situation.

The strength of the present study is that we selected patients in a way that was not done before. We included patients with a presumed LGG that were equally eligible for all three treatment strategies, by using preoperative characteristics typical for a prognostically favorable LGG, and not histopathological diagnosis. Survival was measured from the date of the first diagnostic scan. We consider this design to result in more clinically relevant conclusions than those of earlier studies, since our selection resulted in more unbiased inclusion and, therefore, a more equitable comparison of strategies compared to previous studies.

We observed no difference in OS between early resection and an initial wait-and-scan approach. This suggests that a wait-and-scan strategy can be safely proposed until evident growth, contrast enhancement or clinical deterioration occurs, and that the timing of surgery does not influence the prognosis. How to interpret this result? Similar findings have been found in other cancers with typically long survival times; early prostatectomy did not increase survival as compared to a watchful waiting policy in a large prospective trial in localized prostate cancer with 10 years follow-up [33, 34]. This trial shows that timing of the intervention does not have the impact as expected. Overall survival is not influenced by early intervention as long as the patient is monitored and intervention takes place when necessary. Although our study was not set-up prospectively, the results are comparable. It suggests that in tumors with relatively long overall survival, the relative short timing to treatment intervention is not influencing prognosis. The intrinsic biological behaviour of the tumor (molecular markers) has more impact than the timing of treatment. It also implies that potential morbidity of surgery or biopsy can be safely delayed in these patients and lead to higher quality of life until treatment [35]. On the other hand, surgical techniques like awake craniotomy have been shown to be safe and could also decrease seizure frequency and medication intake in patients with LGG. The data are however not mature yet to give final conclusions, but might already argue that a prospective trial is urgently needed to investigate if surgery can be safely delayed in a subset of presumed LGG patients.

In contrast to early resection versus wait-and-scan, this study shows that biopsy as initial strategy has a negative impact on OS. This observation is in line with that of the Norwegian study [24] and suggests that this strategy should be avoided. It is difficult to explain the significantly shorter OS for the biopsy group compared to the wait-and-scan group. In our cohort, we tried to select patients that were equally eligible for all treatment strategies. Nevertheless, we did observe a higher percentage of astrocytomas in the biopsy group, which may partly explain the poorer prognosis. Alternatively, this difference in histology might be caused by sampling error in the biopsy group. Indeed, in a study examining histological diagnosis in paired biopsy and resection samples, an oligodendroglial component was missed in 50% of the biopsy samples [36]. If this is so, our study implies that biopsy is associated with a less favorable outcome. Moreover, in our multivariate analysis that corrected for histology, a worse prognosis remained for the biopsy group. Nevertheless, it cannot be ruled out that confounding factors that we missed/did not consider might have influenced physicians’ decision to choose for a biopsy procedure and, therefore, biased the selection for patients with poorer prognosis for the biopsy. To be on the safe side we think a biopsy should be avoided when possible. A negative effect of the biopsy itself seems unlikely although an acute inflammatory response induced by biopsies is reported to promote metastasis and proliferation in other types of cancer and recently it was shown in a murine model that reactive astrocytes can potentiate glioma aggressiveness after resection [37–40].

This study has a few limitations. First, this study is retrospective in design. Although our selection criteria aimed to diminish the possible selection and indication bias which comes with such a design, bias is never ruled out completely. Also, the stringent selection criteria that were used resulted in a relatively small cohort size, but they were used to identify those patients in whom an extensive resection is possible according to current standards. Secondly, a longer follow-up is required before definite conclusions can be drawn, as we have not yet reached the median OS in the early resection group. Longer follow-up time is necessary.

Thirdly, the extent of resection also has an impact on OS [15–17, 20, 22, 23]. Perhaps the most important limitation of our study is that the extent of resection was not measured in our cohort, since this might have influenced survival. Moreover, the awake craniotomy procedure that has emerged in glioma surgery, is reported to increase resection percentage and decrease morbidity compared to general anesthesia [41]. In our three treatment groups, the type of anesthetic procedure at the time of resection was chosen based on the best practice at that time. Of the 128 patients that had any resection during follow-up, 56 (43%) were operated with an awake craniotomy procedure and the use of this procedure was equally distributed between the treatment groups. Given this equal distribution and the fact that we selected patients with a lesion eligible for extensive resection, it is unlikely that the extent of resection plays an explanatory role in our results. It can never be ruled out however. Also, of the patients in the early resection group, 20% underwent the actual intervention within 6–12 months after the diagnostic scan. Nevertheless, as the initial choice of treatment by the physician was early resection and the intervention took place without any sign of clinical deterioration, tumor growth or contrast enhancement on MRI scan, we decided not to exclude these patients from the analysis. The sensitivity analysis performed after exclusion of these patients, failed to show different results.

It should be noted that there were imbalances in the treatment and tumor characteristics that were obtained after initial treatment decision, which should be taken into account when interpreting the data. The wait-and-scan group consisted of more high-grade gliomas; this is, however, to be expected since the histological diagnosis in the wait-and-scan group was obtained at a median of 35.4 months after the initial imaging diagnosis. Also, there was an imbalance in chemotherapy administration, showing a lower percentage of patients exposed to chemotherapy in the early resection group. This is possibly explained by the fact that a significant part of the patients in the early resection group had a subsequent wait-and-scan approach after initial resection. It is to be expected that also these patients will receive chemotherapy when follow-up is longer. On the other hand, although median follow-up in the early resection group is shorter, this might suggest that patients in the early resection group are more clinically stable than the other treatment groups. If this is so, we would expect the survival curves to further diverge with longer term follow-up.

Recently the WHO classification of tumors of the central nervous system was updated and now incorporates molecular markers. This new classification outflanks classic histopathological classification in terms of prognosis estimation and can also tailor therapy. Impact of surgery possibly differs between molecular subgroups, but this remains to be investigated. The integration of these markers in our study would be very interesting in the light of the new WHO classification. Unfortunately, the status of these markers was not available for the majority of our population [42, 43]. However, these molecular markers are only determined after resection and should therefore not play a role in the selection and decision criteria of this study. New techniques are now developed to determine the molecular make up of presumed LGG on preoperative MRI’s [44]. This will hopefully lead to optimal treatment strategies of these tumors in the near future, which will also require further analysis of the value of early and of extent of resection in the molecular glioma subtypes .

## Conclusion

Investigation of three different treatment strategies in a clearly defined set of presumed LGG patients who were candidates for extensive resection could not confirm superiority of early resection over wait-and-scan. In agreement with previous studies, biopsy as first treatment strategy seems to be associated with significantly shorter overall survival. Still, this observation is difficult to explain. However, to be on the safe side, we think avoidance of this strategy should be considered when possible. To our knowledge this is the first study to investigate treatment strategies for presumed LGG with this design based on preoperative imaging characteristics, which is highly representative for daily clinical presentation of this patient group. We need longer term follow-up upon final conclusion, but this data highlights prospective data is of vital importance.
